# Evaluating Drivers of the Patient Experience Triangle: Stress, Anxiety, and Frustration

**DOI:** 10.3390/ijerph20075384

**Published:** 2023-04-04

**Authors:** Sumaya Almaazmi, Mecit Can Emre Simsekler, Andreas Henschel, Abroon Qazi, Dounia Marbouh, Rana Adel Mahmoud Ali Luqman

**Affiliations:** 1 Department of Industrial and Systems Engineering, Khalifa University of Science and Technology, Abu Dhabi 127788, United Arab Emirates; 2 Department of Electrical Engineering and Computer Science, Khalifa University of Science and Technology, Abu Dhabi 127788, United Arab Emirates; 3 School of Business Administration, American University Sharjah, Sharjah 26666, United Arab Emirates; 4 Mubadala Healthcare, IPIC Square, Abu Dhabi 45005, United Arab Emirates

**Keywords:** machine learning, patient anxiety, patient experience, patient frustration, patient satisfaction, patient stress, quality, random forest, patient data

## Abstract

Patient experience is a widely used indicator for assessing the quality-of-care process during a patient’s journey in hospital. However, the literature rarely discusses three components: patient stress, anxiety, and frustration. Furthermore, little is known about what drives each component during hospital visits. In order to explore this, we utilized data from a patient experience survey, including patient- and provider-related determinants, that was administered at a local hospital in Abu Dhabi, UAE. A machine-learning-based random forest (RF) algorithm, along with its embedded importance analysis function feature, was used to explore and rank the drivers of patient stress, anxiety, and frustration throughout two stages of the patient journey: registration and consultation. The attribute ‘age’ was identified as the primary patient-related determinant driving patient stress, anxiety, and frustration throughout the registration and consultation stages. In the registration stage, ‘total time taken for registration’ was the key driver of patient stress, whereas ‘courtesy demonstrated by the registration staff in meeting your needs’ was the key driver of anxiety and frustration. In the consultation step, ‘waiting time to see the doctor/physician’ was the key driver of both patient stress and frustration, whereas ‘the doctor/physician was able to explain your symptoms using language that was easy to understand’ was the main driver of anxiety. The RF algorithm provided valuable insights, showing the relative importance of factors affecting patient stress, anxiety, and frustration throughout the registration and consultation stages. Healthcare managers can utilize and allocate resources to improve the overall patient experience during hospital visits based on the importance of patient- and provider-related determinants.

## 1. Introduction

The patient experience is a multi-dimensional concept driven by various patient- and provider-related determinants [1]. The patient experience is defined as “the degree of convergence between the expectations the patients have of ideal care and their perception of the care they really get” [2]. Donabedian defines the patient experience as “the expression of patients judgment” on the quality of care received in all aspects [3], particularly concerning the interpersonal process. Moreover, patient experience is an index of the quality of care provided and is an essential element of patient satisfaction [4].

In many studies, patient satisfaction is an essential and commonly used indicator for measuring healthcare quality. Earlier studies have shown that a patient’s experience has become a crucial indicator of the quality of healthcare services and is used widely at many healthcare institutions [1,5]. For instance, patient experience studies have been classified as a ‘priority’ in quality improvement efforts [2]. Hence, a positive patient experience may result in potential benefits, such as reducing the cost of malpractice and decreasing the length of patient visits and wait time, thereby reducing treatment costs and increasing productivity [6].

Earlier studies have identified several factors, mainly into two categories, that affect the patient experience [1,6]. The first category includes patient-related determinants, including sociodemographic elements, such as gender, age, familial status, profession, and education. The second category includes provider-related determinants, including time, procedure, and the behavioral attributes that patients experience during hospital visits. Furthermore, such experiences could affect patients in different forms, including stress, anxiety, and frustration. However, there is limited research regarding the association between stress, anxiety, frustration, and patient/provider-related determinants.

### 1.1. Patient Stress

Hospitals are places that may affect a patient’s stress levels due to various procedural and behavioral factors [7]. The environmental factors in hospitals can positively or negatively influence patient stress. The literature focuses on the physical and psychological stressors associated with hospitalization, including high noise levels, the absence of natural light, the absence of clocks, and the presence of undesirable and irritating odors [8]. William and colleagues presented an experimental framework that labeled patient stress as a central factor in provider–patient interactions and, eventually, the quality of service and care delivered [9]. Furthermore, they described a model that can be negative or positive that is dependent on the quality of the medical encounter, with a positive correlation between stress management and the quality of care [9].

High levels of stress hormones, including cortisol and catecholamines, are released when a patient is stressed. Such hormones are measured via laboratory tests using samples from blood, saliva, and urine that may be seen as invasive/uncomfortable and lengthy by the patient, especially when the results exceed the turnaround time or when the blood sample collection is not taken appropriately by a well-trained nurse, resulting in further increases in the patient’s level of stress. On the other hand, non-invasive, immediate measurements for stress are available [10], such as changes in heart rate (HR) [11], blood pressure (BP) [12], pupil diameter (PD), breathing pattern [13], galvanic skin response (GSR) [14], voice intonation [15], and body pose [16]. Therefore, physiological features (i.e., heart rate or skin conductivity) and physical features (i.e., facial expressions, voice intonation, body poses, and gestures) enable such methods and can be used to objectively model stress [10]. When stress is well managed, the quality of the medical encounter is good, and this reflects positively on patient satisfaction, compliance, and health outcomes [17]. Hence, the patient is more likely to perceive the interactions with the provider as fulfilling and rewarding. However, when stress is poorly managed, the quality of interactions with the patient and patient-centered communication is negatively impacted, resulting in a negative patient experience [18].

### 1.2. Patient Anxiety

Anxiety is defined as a “feeling of nervousness or worry about something that is happening or might happen in the future” [9]. The fear of the unknown, long-term sickness, and dying contribute to patient anxiety [9]. Moreover, anxiety has several symptoms, including a lack of concentration, tiredness/fatigue, and muscular tension. Anxiety can affect everyone, whether temporary or persistent, leading to violent behavior, and making it difficult to undergo pain management or to achieve positive health outcomes [19]. Anxiety is not routinely assessed in hospitalized patients, and failure to identify and treat anxious patients makes them more vulnerable to the adverse effects of anxiety, including higher complication rates [20]. The causes of complication rates among anxious patients are anxiety-related behaviors, such as a poor diet, smoking, poor compliance with a treatment plan, and an inactive lifestyle [21].

There are several ways to assess and measure anxiety in a hospital setting. For instance, patients can self-report their symptoms of anxiety through a survey [22]. Furthermore, surveys with physiological variables, such as heart rate, nervous and muscular activity, and palmar sweating, have been used to measure patient anxiety [23].

### 1.3. Patient Frustration

Frustration and the notion of stress are used interchangeably, although there is an essential distinction between the two. Stress presents from an overload of mental, physical, or emotional build-up and does not necessarily coincide with frustration. Frustration is accompanied by a psychological response. There are two ways to assess frustration: self-reporting and physiological measures [24]. The self-reporting method consists of collecting patients’ self-reported data and asking them to rate their frustration levels. The other method assesses physiological measures in order to measure frustration, since the feeling of frustration is accompanied by physiological changes. This method is potentially less biased and is more objective compared to the self-reporting method. Frustration is the feeling of being distraught, annoyed, and upset because of the inability to achieve or change a situation. It is acknowledged as an unpleasant and disturbing negative feeling for patients, especially those with chronic conditions. For instance, a recent study showed that 97% of clinical patients suffering from back pain or post-hepatic neuralgia experienced severe frustration levels, ranking higher than seven on a ten-point scale [25].

Frustration is associated with anger, fear, and discomfort and is ranked higher than other negative pain-related emotions, including despair, fear, anxiety, and distress. Frustration is a precursor to anger, affecting patient safety and wellbeing [25]. Wade and colleagues also showed that frustration played a substantial role in the pain associated with discomfort, and suggested that frustration-specific therapy strategies could be helpful [26].

Despite the number of patient experience studies, the literature lacks investigations of the triangle of stress, anxiety, and frustration, and their drivers have not been discussed in the same study setting [27]. Thus, considering the concept of a multi-dimensional patient experience, with possible interactions between each other and their association with the patient experience, these factors are not well understood in this research context and have not been well explored using the available tools. Concurrently, machine learning (ML) algorithms, such as tree-based ensemble learning algorithms, may possess higher prediction capabilities and feature important analyses in order to evaluate the patient experience throughout the patient journey [28].

Many studies have addressed patient experience; however, the dimensions of stress, anxiety, and frustration and their corresponding drivers remain debatable, with little data available in the literature to understand their importance and potential effects on the patient experience. It is clear that there is no standardized framework for measuring the patient experience around the dimensions of; stress, anxiety, and frustration, which makes the concept seem complicated and, as a result, more challenging to determine the main drivers.

Measuring patient stress, anxiety, and frustration is not easy, as measuring tools are numerous and vary across studies [1]. Thus, a unified instrument measuring these dimensions can help healthcare managers analyze the drivers and differences. Additionally, there is a lack of standardized methodological tools and models to measure the patient experience [29].

This study aims to explore the drivers of patient stress, anxiety, and frustration by ranking their importance throughout two patient journey stages, namely registration and consultation. In order to achieve this, we apply an ML-based algorithm, Random Forest (RF), which is a reliable classifier with an embedded feature importance analysis. RF consists of many decision trees (DT) trained using different parts of the training dataset. In order to classify a new sample, the input vector of that sample is needed to pass down with each DT of the forest. Furthermore, each DT considers a different part of that input vector and gives a classification outcome. The forest then forecasts the future based on most of the predictions rather than relying on a single decision tree [28]. Earlier studies show that RF is highly accurate in shorter training times while decreasing overfitting risks [4]. In this study, RF was selected in order to identify the key drivers and to capture any relationship throughout the patient journey at the hospital.

## 2. Materials and Methods

### 2.1. Data Source

Patient experience surveys were administered at a local hospital in Abu Dhabi, UAE. The survey focused on two stages throughout the patient journey: a non-clinical process, “Registration”, and the clinical process, “Consultation”. The survey covered four sections containing questions, including (1) patient socio-demographics (Table 1), (2) registration-specific questions (Table 2), (3) consultation-specific questions (Table 3), and (4) questions on the patients’ outcome variables of stress, anxiety, and frustration. Registration- and consultation-specific questions included factors related to (i) time, (ii) behavior, and (iii) procedures that played a role during their journey at the hospital.

The questions in the survey, including those in the categories mentioned above, were scored using a 5-point Likert scale (‘not at all’, ‘not’, ‘neutral’, ‘somewhat’, and ‘extremely’). There were 411 survey respondents. Table 1 presents the sociodemographic attributes that were considered patient-related determinants affecting their experience in their hospital visit.

In the non-clinical registration stage, ten questions were asked, including the registration staff’s behavior, time to complete the registration process, and the procedures followed during the registration stage. Table 2 presents the questions related to the registration stage, the question type, and their codes.

In the clinical consultation stage, 16 questions were covered, assessing how the doctor behaved when communicating with the patient, the time taken to see the doctor or the time spent with the doctor, and the procedures followed during this stage. Table 3 presents the questions, along with the type of question and their codes.

### 2.2. Procedure

The RF algorithm was built using Python as the programming language. Introduced by Breiman [30], RF is a highly effective algorithm that is utilized for classification and regression models [31], such that it is a collection of unpruned decision trees that are used when working with large datasets and multiple input variables [32].

The RF model is a classifier that consists of many decision trees [33]. These trees perform a search via a large number of possible binary splits for every feature in order to determine the most optimal split for each node. RF uses bagging to generate a training set for each tree. Each node is divided based on the superior split among a subset of randomly chosen predictors at a node. Due to the random exploration of features, RF lends itself well to feature selection and the measurement of feature importance [34]. Compared to other classifiers, the RF algorithm has also proven to have a solid performance and is known for its substantial robustness in cases of overfitting [4]. RF has mainly four parameters: (i) the maximum depth of each tree in the forest; (ii) the number of trees in the forest; (iii) the minimum number of samples needed before splitting an internal leaf node; and (iv) the minimum number of samples needed at a leaf node. It has also been successfully applied in earlier patient-experience-related studies [28,32,35].

The models were created for the two stages of the patient journey: registration and consultation. There were three outcome variables: overall stress, anxiety, and frustration. There were several explanatory variables in each registration and consultation model. Each model followed the same training and testing pathways, optimizing the hyper-parameters presented (see Table 4) in order to support prediction accuracy.

The training set was divided into five folds. Each time, the classifier produced the model by utilizing the first four folds (k − 1) and tests on the remaining fold (i.e., the fifth fold) for cross-validation. For the next iteration, the model was trained on the first, second, third, and fifth folds and was evaluated on the fourth fold. This process was repeated three more times so that each fold was eventually trained [36]. The final accuracy score of the model was the average accuracy of the five folds.

In the next stage, the randomized search function repeated the steps by taking a new combination of hyper-parameters. Finally, after completing this process and examining all the potential hyper-parameter combinations, the optimal model with the best accuracy score was achieved. Equation 1, presented below, was used to calculate the prediction accuracy in the case of the absence of class imbalance [28,37].

(1) $$Accuracy=\frac{number\;of\;correct\;predictions}{total\;number\;of\;records}$$

## 3. Results

Table 5 presents the responses to the patient-related determinant questions in percentages. The results show that 89.1% of the participants were UAE nationals, whereas 10.9% were of different nationalities. Similarly, 54.3% of the participants were men, and 34.6% of admitted patients were over 65 years of age. Established patients, who visited the hospital in the past, comprised 21.65% of respondents compared to new patients, 78.35%.

The hyper-parameter values that were used are presented in Table 6, along with the accuracy score of the testing sets. The hyper-parameter values that were used before tuning represent the commonly used values in Python [38]. The prediction accuracy of the RF algorithm was improved by tuning the hyper-parameters. For instance, the average accuracy increased significantly for overall stress and frustration in the consultation process in the two RF models.

**Figure 1:**
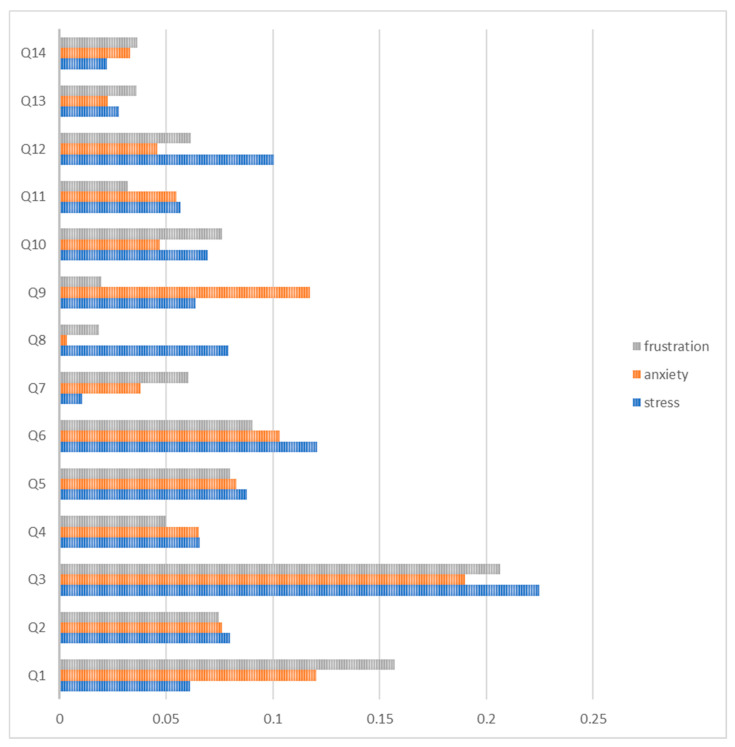
Relative importance summary of the RF model for overall stress, anxiety, and frustration throughout the registration step.

Following training and optimization of the model, a feature importance analysis was conducted. The first model represents overall stress, anxiety, and frustration during registration, as illustrated in Figure 1. The x-axis represents the “feature importance” score, whereas the y-axis represents all the “explanatory variables”. The “explanatory variables” represent the survey questions relevant to the Registration step and are ranked based on their importance. For stress, it is clear from Figure 1 that ‘Q3′ (22.48%), which represents age, is the top-ranked variable compared to the other “explanatory variables”. This is followed by ‘Q6′ (12.07%), which is the ‘total time taken for registration’ and ‘Q12′ (10.04%), which represents the ‘knowledge of the registration staff while handling the registration process’. The least important feature in this model is ‘Q7′ (1.05%), representing the ‘approachable and smiling manner of the registration staff’.

For anxiety, the top-ranked variable here is ‘Q3′ (18.99%), followed by ‘Q1′ (12.02%), which also belongs to the patient-related determinants category, representing nationality. The third determinant is ‘Q9′ (11.75%), which refers to the survey question ‘courtesy demonstrated by the registration staff in meeting your needs’ and belongs to provider-related determinants. The variables ‘Q8′ (0.34%) and ‘Q13′ (2.26%) refer to the survey questions ‘professionalism of the registration staff’s appearance’ and ‘sensitivity of registration staff to confidential information’, respectively. These had the lowest scores in the feature importance plot.

For frustration, interestingly, the ‘Q3′ (20.64%) variable was also the top-ranked variable in this model, followed by the variables ‘Q1′ (15.69%) and ‘Q6′ (9.06%), which refers to the survey question ‘total time taken for registration’. The ‘Q8′ (1.87%) and ‘Q9′ (1.95%) variables, which correspond to the questions ‘professionalism of the registration staff’s appearance’ and ‘courtesy demonstrated by the registration staff in meeting your needs’, respectively, both had the lowest values in the feature importance plot.

**Figure 2:**
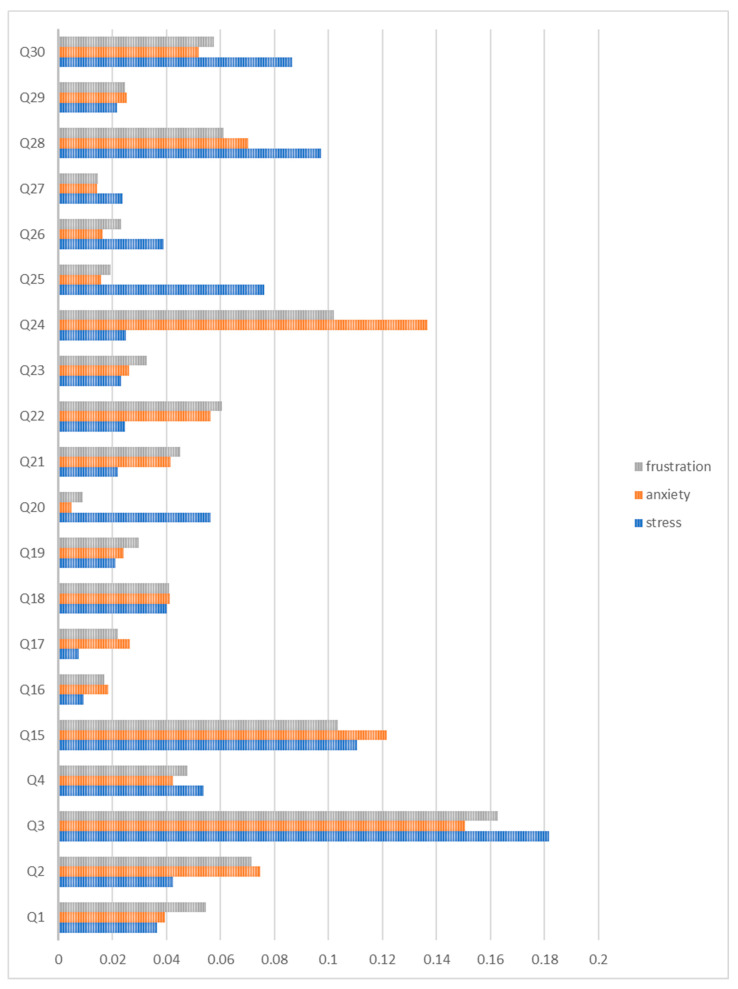
Relative importance summary of the RF model for overall stress, anxiety, and frustration throughout the consultation step.

The next stage to be analyzed in a patient’s journey in the hospital was the clinical process step: the consultation process. Figure 2 demonstrates the essential results of the model, including the overall stress, anxiety, and frustration. For stress, ‘Q3′ (18.17%), which belongs to the category of patient-related determinants, has the highest score in the model, which is the same as all the previous models. The next most important variable was ‘Q15′ (11.05%), which refers to the question ‘waiting time to see the doctor/physician’. The third most important variable in this model was ‘Q28′ (9.73%), which corresponds to the ‘doctor/physician’s explanation of the next steps in treatment (e.g., tests, medications, etc.). Furthermore, ‘Q17′ (0.76%) and ‘Q16′ (0.93%) contributed the least to overall stress in this model. Q17 and Q16 represent ‘productive use of your time with the doctor/physician’ and ‘total time spent with the doctor/physician’, respectively.

Similarly, the dominant variable affecting overall anxiety in this model was ‘Q3′ (15.05%), as for all the previous models. The second variable was ‘Q24′ (13.65%), which corresponds to the survey question ‘the doctor/physician was able to explain your symptoms using language that was easy to understand’, belonging to the provider-related determinants category. The third variable affecting overall anxiety was ‘Q15′ (12.16%), which corresponds to the question ‘waiting time to see the doctor/physician’. Unlike other models, patient-related determinants do not comprise the top three drivers in this model; ‘Q3′, the leading driver, was the only patient-related determinant in this model. The variables that contribute least to this model were ‘Q20′ (0.49%) and ‘Q27′ (1.42%), which correspond to ‘courtesy demonstrated by the doctor/physician to your needs’ and ‘the levels of hygiene and cleanliness in the consultation room’, respectively.

For overall frustration during the consultation process, the top-ranked variable was ‘Q3′ (16.26%), which belongs to the patient-related determinants category, followed by ‘Q15′ (10.34%), which refers to ‘waiting time to see the doctor/physician’. This is followed by ‘Q24′ (10.2%), corresponding to ‘the doctor/physician was able to explain your symptoms using language that was easy to understand’. As for the previous model, the variables that contributed least to this model were ‘Q20′ (0.94%) and ‘Q27′ (1.46%), which correspond to ‘courtesy demonstrated by the doctor/physician to your needs’ and ‘the levels of hygiene and cleanliness in the consultation room’, respectively.

## 4. Discussion

The RF algorithm was developed in this study in order to identify the most important features influencing patient stress, anxiety, and frustration in two stages of the patient journey in a local hospital: registration and consultation. Interestingly, the most influential driver in all our models was the variable ‘Q3′, representing age. Thus, the goal of this research was accomplished by determining the main determinants of patient stress, anxiety, and frustration.

In all the models (overall stress, anxiety, and frustration for both the registration and consultation stages), ‘Q3′, which indicates patient age and belongs to the category of patient-related determinants, was the most influential attribute amongst all the other factors associated with the demographic questions. This finding is in line with earlier studies, as age is highly associated with the level of stress of hospitalized patients [39]. Another study was conducted in order to investigate the relationship between patients’ sociodemographic characteristics and their stress levels and revealed that age influenced patients’ stress levels. During hospitalization, younger patients experienced higher stress levels than older patients [40]. Additionally, Gullich and colleagues [41] identified the importance of age for patients’ anxiety levels. Thus, age can cause a negative change in a patient’s anxiety levels; younger patients were shown to be more anxious during their hospital visits compared to older patients. Nevertheless, there is insufficient literature to support the correlations associating age with a level of frustration.

Concurrently, in the clinical process models (consultation process), the second most crucial driver for the overall stress model was ‘Q15′, which corresponds to the survey question ‘waiting time to see the doctor/physician’. This belongs to the provider-related determinant category. In the overall anxiety model, the question ‘waiting time to see the doctor/physician’ was the third most important driver when using the RF algorithm for the analysis.

For the overall frustration model, the same question (Q15) was the second most important driver when using the RF algorithm for the analysis. Thus, these findings indicate the importance of time-related questions for patients’ stress, anxiety, and frustration levels, which is consistent with other studies, where long wait times have negatively impacted the patients’ experiences [42]. Moreover, unnecessary wait times, due to inefficient hospital processes or overcrowding during peak hours, may increase patients’ stress levels. Therefore, the level of patient satisfaction is correlated with the duration of time spent waiting [43]. Furthermore, the literature shows that waiting time at emergency departments (ED) increases anxiety in patients since they tend to think about negative scenarios while waiting [44]. It should be noted that patients admitted to the ED are likely to feel unwell and fatigued, due to the long time spent waiting to be seen, and they leave the ED, which adds to the stress and anxiety of the patient.

The third most important driver for the consultation process in the overall stress and anxiety models is ‘doctor/physician’s explanation of the next steps in treatment (e.g., tests and medications)’, which is a procedure-related question. Similarly, ‘knowledge of the registration staff whilst handling the registration process’ was the third top driver for the overall stress model in the registration step when using the RF. The drivers related to procedure questions correlate with the technical care determinant. Procedure-related questions reflect health professionals’ competency, efficiency, and ability to provide pleasant service [1]. A study conducted at a local hospital in Malaysia showed that not knowing doctors’ or nurses’ plans for treatment was a stress driver for patients [39]. Additionally, uncertainty about pre-procedure steps exposes patients to higher levels of anxiety [45].

The findings of this study can be summarized into two main observations. The first observation is that patient-related determinants are dominant in overall stress, overall anxiety, and overall frustration models for the registration step. ‘Q3′, ‘Q1′, and ‘Q2′, which belong to the demographic question type, were the top drivers in these models based on their high feature importance scores. The attribute ‘Q3′ was the first driver in all the models. Therefore, this finding shows the importance of patient-related determinants and their effects on a patient’s stress, anxiety, and frustration levels. Second, the time-related question type, which belongs to the provider-related determinants category, was the most significant question in the overall stress, anxiety, and frustration models for the consultation process. The most common variable in these three models was ‘waiting time to see the doctor/physician’, emphasizing the importance of waiting time for patients.

Our results have shown limitations that may offer future research opportunities. For instance, this study is based on data from a specific patient population from a single hospital and country; therefore, generalizability to other healthcare settings may be limited. Although the survey covered a comprehensive list of patient- and provider-related determinants, different dimensions, such as culture, language, and prior health conditions, could affect the patient experience during the hospital visit. Future research may also benefit from evaluating different ML algorithms in order to identify possible improvements in prediction capabilities and relative importance analyses.

## 5. Conclusions

In this study, the RF algorithm was developed in order to explore the main drivers of patient stress, anxiety, and frustration throughout two stages of patient journey in the hospital the (registration and consultation processes). Using the algorithm, the remarkable limitations in the current literature regarding the determinants of a patient’s stress, anxiety, and frustration were addressed.

Based on its high feature importance score, the leading driver of patients’ stress, anxiety, and frustration in all the models was age, which is a patient-related determinant. In the registration process, ‘total time taken for registration’ was the key driver of patient stress, whereas ‘courtesy demonstrated by the registration staff in meeting your needs’ was the key driver of anxiety and frustration using the RF model. In the consultation process, ‘waiting time to see the doctor/physician’ was the key driver of both patient stress and frustration, whereas ‘the doctor/physician was able to explain your symptoms using language that was easy to understand’ was the main driver of anxiety using the RF model.

Another contribution of this study is the development of a novel ML algorithm using data from patient experience surveys. The results can provide hospitals with significant insights into the different drivers affecting patient stress, anxiety, and frustration. In addition, our findings can aid hospitals in allocating and prioritizing their resources based on the importance of different factors.

## Figures and Tables

**Table 1:** Demographic questions in the survey.

Code	Questions	Type of Question
Q1	What is your nationality?	Demographic
Q2	Record your gender.	Demographic
Q3	Can you please tell me your age?	Demographic
Q4	Please indicate the below: new patient or established patient?	Demographic

**Table 2:** Registration-specific questions in the survey.

Code	Questions	Type of Question
Q5	Time taken upon arrival to acknowledge you at the registration desk	Time
Q6	Total time taken for registration	Time
Q7	Approachable and smiling manner of the registration staff	Behavior
Q8	Professionalism of the registration staff’s appearance	Behavior
Q9	Courtesy demonstrated by the registration staff in meeting your needs	Behavior
Q10	Attentiveness and knowledge of the registration staff while listening to your queries	Behavior
Q11	Proactive behavior of the registration staff in explaining the next steps	Behavior
Q12	Knowledge of the registration staff whilst handling the registration process	Procedure
Q13	Sensitivity of registration staff to confidential information	Procedure
Q14	Explanation of next steps in the process and time expectations	Procedure

**Table 3:** Consultation-specific questions in the survey.

Code	Questions	Type of Question
Q15	Waiting time to see the doctor/physician	Time
Q16	Total time spent with the doctor/physician	Time
Q17	Productive use of your time with the doctor/physician	Time
Q18	Approachable and smiling manner of the doctor/physician	Behavior
Q19	Professionalism of the doctor/physician’s appearance	Behavior
Q20	Courtesy demonstrated by the doctor/physician to your needs	Behavior
Q21	Attentiveness and knowledge of the doctor/physician while listening to your queries	Behavior
Q22	Ability of the doctor/physician to make you feel comfortable and relaxed	Behavior
Q23	Professional competency demonstrated by the doctor/physician	Behavior
Q24	The doctor/physician was able to explain your symptoms using language that was easy to understand	Behavior
Q25	Doctor’s level of awareness of previously collected data (history and physical)	Procedure
Q26	Privacy of the consultation location	Procedure
Q27	The levels of hygiene and cleanliness in the consultation room	Procedure
Q28	Doctor/physician’s explanation of the next steps in treatment (e.g., tests, medications, etc.)	Procedure
Q29	Doctor/physician being informed of all aspects of your condition	Procedure
Q30	Nurse’s and doctor’s consistency in the information they shared with you	Procedure

**Table 4:** Parameter search space in grid search analysis.

Parameter	Range
max_depth (maximum depth of the tree)	[3; 4; 5; 6; 7; 8; 9; 10; 12; 15]
min_samples_leaf (minimum number of data points allowed in a leaf node)	[1; 2; 3; 4; 5; 6; 7; 8; 9; 10; 15]
min_samples_split (minimum number of data points in a node before the node is split)	[2; 3; 4; 5; 6; 8; 10; 15]
n_estimator (number of trees in the forest)	[100; 150; 200; 250; 300; 400; 500; 600; 800; 1000; 1200]

**Table 5:** Patient-related determinants.

Patient-Related Determinants		No. of Observation (%)
Q1	Locals	366 (89.1%)
	Foreigners	45 (10.9%)
Q2	Male	223 (54.3%)
	Female	188 (45.7%)
Q3	Age group 1 (youngest)	30 (7.3%)
	Age group 2	94 (22.9%)
	Age group 3	91 (22.1%)
	Age group 4	142 (34.6%)
	Age group 5	52 (12.7%)
	Age group 6 (oldest)	2 (0.5%)
Q4	New patient	322 (78.4%)
	Established patient	89 (21.7%)

**Table 6:** Machine learning hyper-parameter values and accuracy rates.

Hyper-Parameters	Registration Process					
	Overall Stress		Overall Anxiety		Overall Frustration	
	BT	AT	BT	AT	BT	AT
	Consultation Process					
	Overall Stress		Overall Anxiety		Overall Frustration	
	BT	AT	BT	AT	BT	AT
max_depth	None	10	None	10	None	10
min_sample_leaf	1	1	1	1	1	2
min_sample_split	2	5	2	5	2	5
n_estimators	10	2000	10	2000	10	1600
Accuracy	0.86	0.89	0.81	0.86	0.82	0.86
max_depth	None	40	None	10	None	10
min_sample_leaf	1	2	1	4	1	2
min_sample_split	2	10	2	2	2	5
n_estimators	10	400	10	1600	10	1000
**Accuracy**	0.86	0.92	0.81	0.83	0.7	0.83

BT: before tuning; AT: after tuning.

## Data Availability

Contact the corresponding author who can connect interested parties to the data holder.
